# Supplementary material for: Effect of Particle Strength on SiCp/Al Composite Properties with Network Architecture Design
Source: Materials (Basel). 2024 Jan 26;17(3):597. doi: 10.3390/ma17030597 (PMC10856433; doi:10.3390/ma17030597)
Supplement: Supplementary file 1 [file materials-17-00597-s001.zip › materials-2624208-supplementary.pdf]

Supplementary Materials

The effect of particle strength on SiCp/Al composite properties with network architecture design

Xiang Gao[1,2], Xiaonan Lu[1,2], Xuexi Zhang[3]*, Mingfang Qian[3], Aibin Li[3], Lin Geng[3], Huan Wang[2], Cheng Liu[2], Wenting Ouyang[2], and Hua-Xin Peng[1,2]*

[1] Ningbo Innovation Center, Zhejiang University, Ningbo 315100, China

[2] Institute for Composites Science Innovation (InCSI), School of Materials Science and Engineering, Zhejiang University, Hangzhou 300027, China

[3] School of Materials Science and Engineering, Harbin Institute of Technology, Harbin 150001, China

*Corresponding author. E-mail address: hxpengwork@zju.edu.cn (HXP), xxzhang@hit.edu.cn (XXZ).

Model, simulation results and network architecture example for metal-matrix composites

In the models, the matrix was 6061 aluminum alloy subjected to T6 heat treatment, with density 2.70 g/cm^3, Young's modulus 68.9 GPa, Poisson's ratio 0.33 and yield strength 269.1 MPa (Figure S1). Ductile failure behavior of matrix was expressed by 'ductile damage criterion', in which cracks initiate when equivalent plastic strain $\varepsilon^p{}_f$ equals to 0.11 (determined by the experimental stress-strain curve in Figure S1).

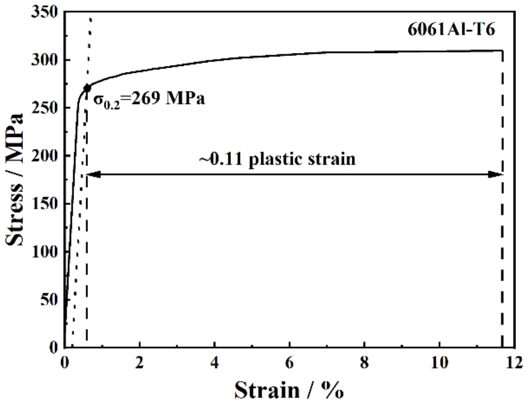

Figure S1: Experimental stress–strain curve of the 6061 Al alloy showing that the alloy exhibited a yield strength of 269 MPa.

The progressive damage degradation behavior is presented in the Figure S2 [1], which is given by:

$$E = (1 - D)\overline{E} \tag{S1}$$

where D is the stiffness degradation variable, E is the Young`s modulus with degradation to represent the stiffness, $\overline{E}$ is the undamaged stiffness tensor. So the stress degradation can be expressed by:

$$\sigma = \overline{\sigma} - D\overline{\sigma} = (1 - D)\overline{\sigma} \tag{S2}$$

where σ is stress tensor with degradation and $\overline{\sigma}$ is the undamaged stress tensor.

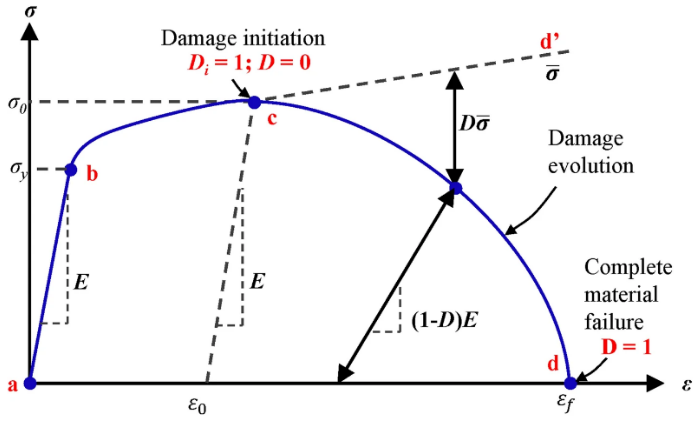

Figure S2 Stress-strain curve with progressive damage degradation [1].

The previous observation on fracture surface showed matrix attached on the particle surface (Figure S3) [2,3]. This reveals that a strong cohesion state of SiC/Al interface.

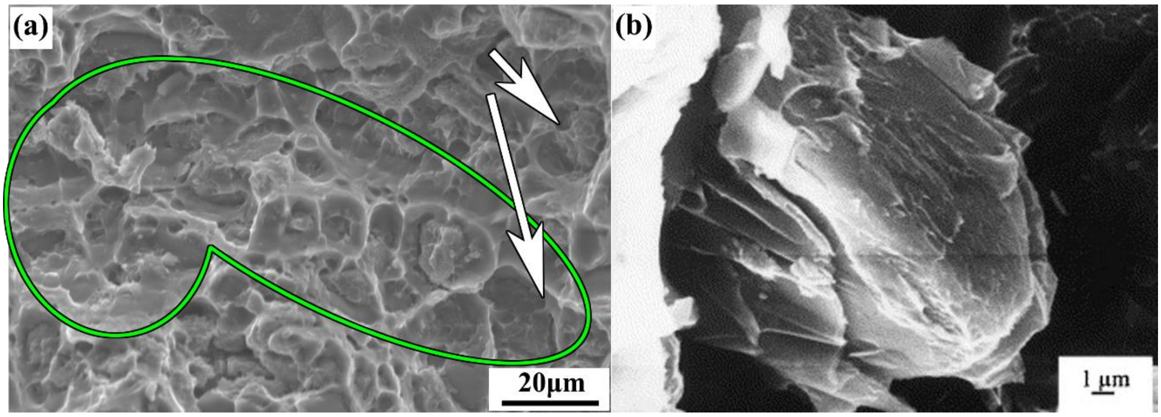

Figure S3 Retained matrix on SiC surface in SiCp/Al composites with network [2] and homogeneous [3] distribution.

The finite element (FE) model was validated in our previous work [4]. The predicted stress-strain curves and mechanical properties of 10, 15 and 20vol.% SiCp/6061Al composites are close to the experimental counterparts [4-6] (Figure S4, Table S1). In addition, the stability of the models was verified by comparing simulation results of three 10vol.% SiCp/6061Al geometries [7] (Figure S5). Therefore, our numerical method is reliable to predict deformation and fracture behavior of SiC/Al composites.

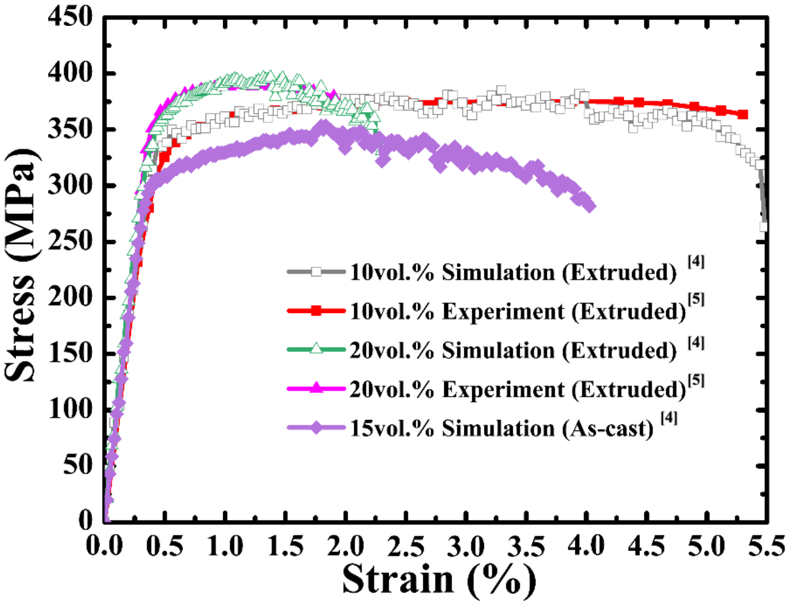

Figure S4 Tensile stress-strain curves comparison between simulation [4] and experiment [5] results.

Table S1 Mechanical properties of the as-cast and extruded SiCp/6061Al composites by experiment and simulation [4-6].

Materials	Method	E (GPa)	$\sigma_{0.2}$ (MPa)	σ_{UTS} (MPa)	ε_f
10vol.% SiCp/6061Al (Extruded)	Simulation [4]	86	345	382	5.4
	Experiment [5]	84	342	375	5.3
15vol.% SiCp/6061Al (As-cast)	Simulation [4]	96	318	353	-
	Experiment [6]	95	320	-	-
20vol.% SiCp/6061Al (Extruded)	Simulation [4]	100	371	396	2.3
	Experiment [5]	97	378	388	1.9

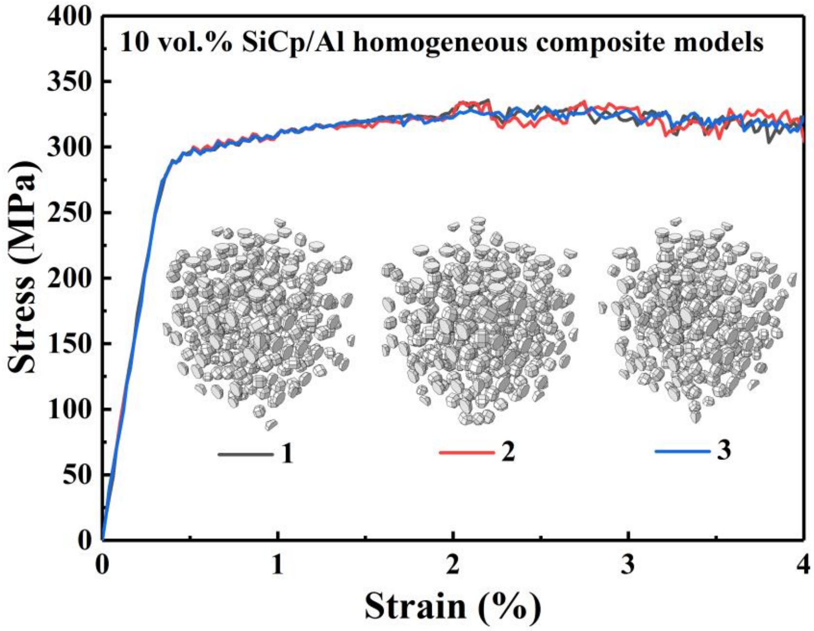

Figure S5 The stress-strain curves of 10 vol.% SiCp/6061Al homogeneous composites with different SiCp distributions [7].

In our previous work, effect of particle size ratio (PSR) was investigated in network SiCp/Al composite [7]. High PSR presented increased modulus due to improved load bearing capability of SiC (see Figure S6).

a)

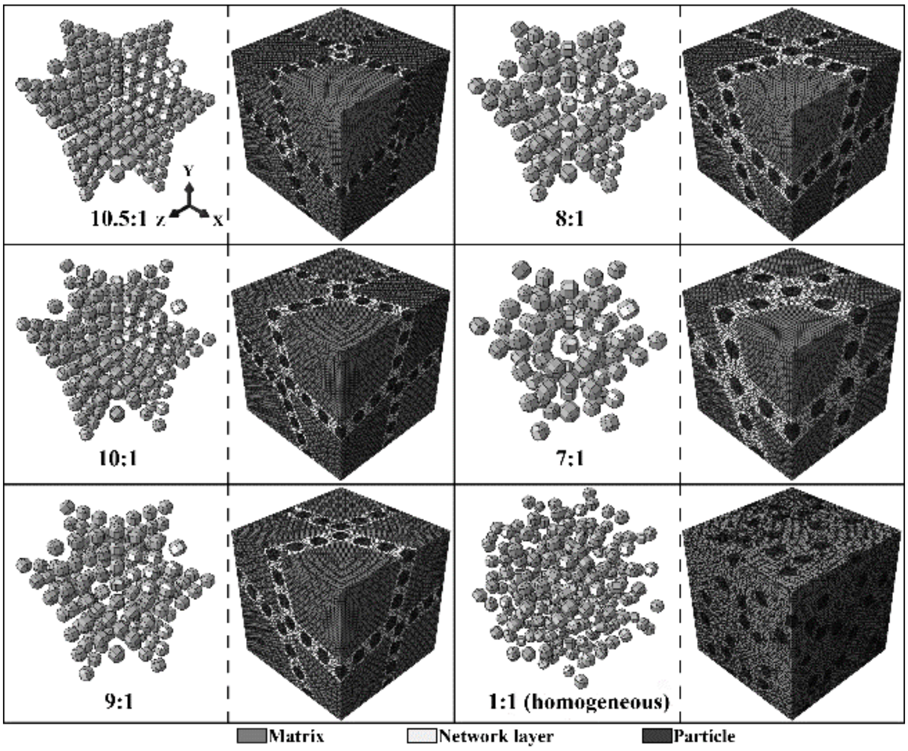

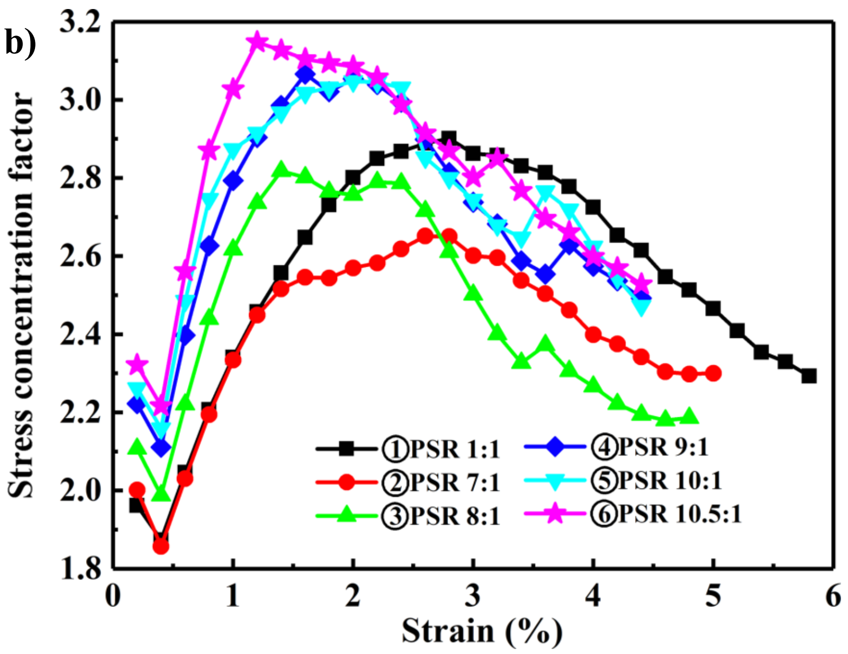

Figure S6 Effect of particle size ratio (PSR) on load bearing capability of reinforcement [7]: (a) geometry and mesh with various of PSR; (b) stress concentration factors evolution curves.

Lee *et al* [8] designed a network architecture, whereas a reduced modulus was shown since almost all network layers were 45° or 90° to load (see Figure S7)

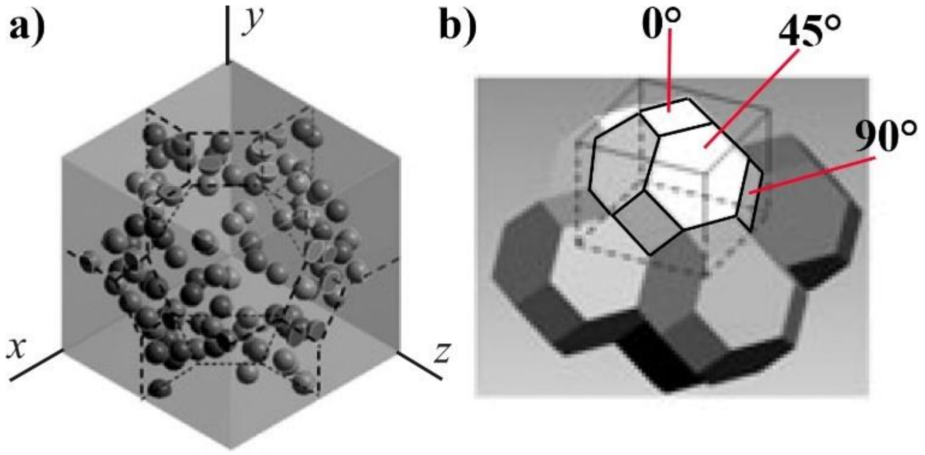

Figure S7 TiCp/Al geometry model with (a) network architecture and (b) network layer orientation [8].

SiCp/Al composites with laminated [9], bar-liked [10] and ring-liked [11] architectures have been successfully prepared (Figure S8). In these composites, the particle aligned parallel to load direction.

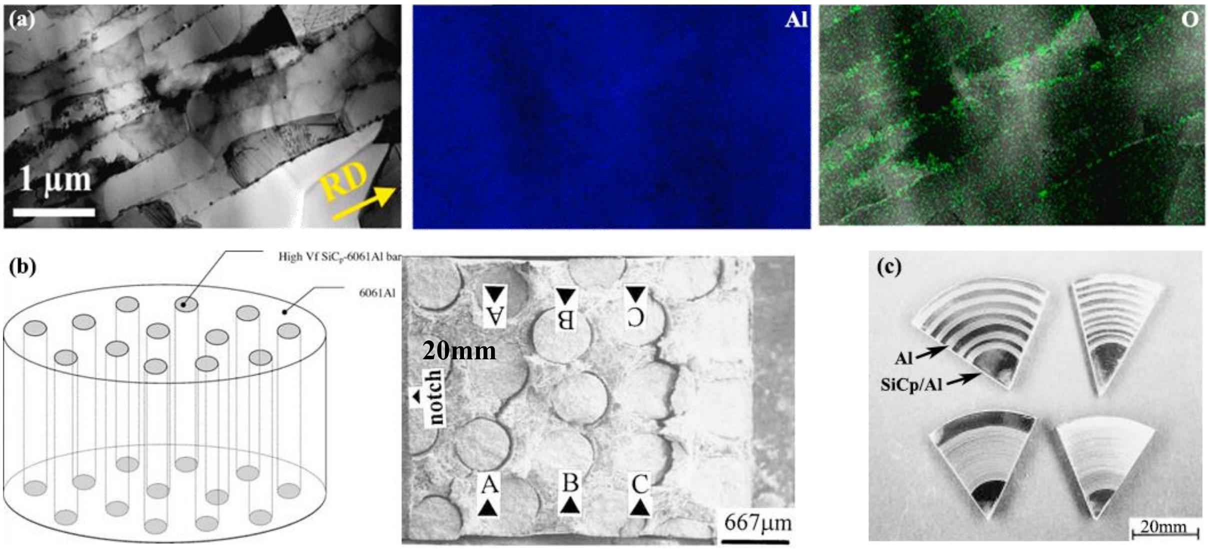

Figure S8 The MMCs with (a) laminated [9], (b) bar-liked [10] and (c) ring-liked [11] structures.

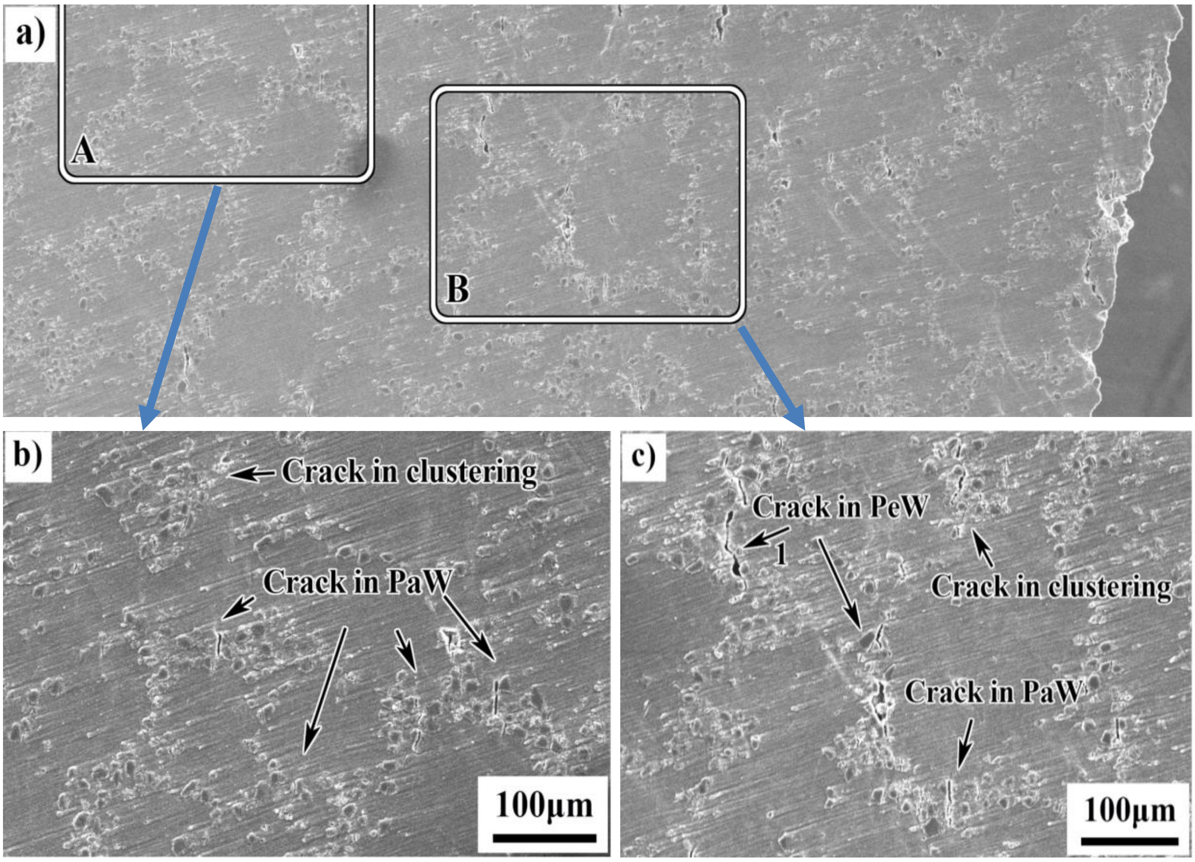

Figure S9 Crack initiation behavior in network SiCp/6061Al composite [2].

References

[1] Gopinath, K.; Narayanamurthy, V.; Khaderi, S.N.; Rao, Y.V.D. Determination of Parameters for Johnson-Cook Dynamic Constitutive and Damage Models for E250 Structural Steel and Experimental Validations. J. Mater. Eng. Perform. 2023. https://doi.org/10.1007/s11665-023-08733-4.

[2] Gao, X.; Zhang, X.X.; Qian, M.F.; Li, A.B.; Geng, L.; Peng, H-X. Fracture behaviour of SiCp/Al composites with network architecture. Materialia 2020, 12, 100725. https://doi.org/10.1016/j.mtla.2020.100725.

[3] Ureña, A.; Escalera, M.D.; Gil, L. Influence of interface reactions on fracture mechanisms in TIG arc-welded aluminium matrix composites. Compos. Sci. Technol. 2000, 60, 613–622. https://doi.org/10.1016/S0266-3538(99)00168-2.

[4] Gao, X.; Zhang, X.; Geng, L. Strengthening and fracture behaviors in SiCp/Al composites with network particle distribution architecture. Mater. Sci. Eng. A 2019, 740–741, 353–362. https://doi.org/10.1016/j.msea.2018.10.105.

[5] Shin, C.S.; Huang, J.C. Effect of temper, specimen orientation and test temperature on the tensile and fatigue properties of SiC particles reinforced PM 6061 Al alloy. Int. J. Fatigue 2010, 32, 1573–1581. https://doi.org/10.1016/j.ijfatigue.2010.02.015.

[6] Hilfi, H.; Brar, N.S. Evaluation of Johnson-Cook model constants for aluminum based particulate metal matrix composites. AIP Conf. Proc. 1996, 370, 559–562. https://doi.org/10.1063/1.50657.

[7] Gao, X.; Zhang, X.X.; Li, A.B. Numerical study on mechanical properties of quasi-continuous SiCp/Al network composites with various particle size ratios (PSRs). Int. J. Appl. Mech. 2019, 11, 1950065. https://doi.org/10.1142/S1758825119500650.

[8] Lee, W.J.; Kim, Y.J.; Kang, N.H.; Park, I.M.; Park, Y.H. Finite-element modeling of the particle clustering effect in a pow-der-metallurgy-processed ceramic-particle-reinforced metal matrix composite on its mechanical properties. Mech. Compos. Mater. 2011, 46, 639–648. https://doi.org/10.1007/s11029-011-9177-y.

[9] Sadeghi, B.; Cavaliere, P.; Balog, M.; Pruncu, C.I.; Shabani, A. Microstructure dependent dislocation density evolution in mi-cro-macro rolled Al2O3/Al laminated composite. Mater. Sci. Eng. A 2022, 830, 142317. https://doi.org/10.1016/j.msea.2021.142317.

[10] Qin, S.Y.; Zhang, G.D. Analyses on fracture characteristics of SiCp-6061Al/6061Al composites extruded by different ratios. J. Mater. Sci. 2002, 37, 879–883. https://doi.org/10.1023/A:1013820805015.

[11] McLelland, A.R.A.; Atkinson, H.V.; Anderson, P.R.G. Thixoforming of a novel layered metal matrix composite. Mater. Sci. Technol. 1999, 15, 939–945. https://doi.org/10.1179/026708399101506616.
